# The Moonlighting Function of Soybean Disordered Methyl-CpG-Binding Domain 10c Protein

**DOI:** 10.3390/ijms24108677

**Published:** 2023-05-12

**Authors:** Yanling Li, Jiawei Qin, Menglu Chen, Nan Sun, Fangmei Tan, Hua Zhang, Yongdong Zou, Vladimir N. Uversky, Yun Liu

**Affiliations:** 1 Guangdong Provincial Key Laboratory for Plant Epigenetics, Shenzhen Key Laboratory of Microbial Genetic Engineering, College of Life Sciences and Oceanography, Shenzhen University, Shenzhen 518060, China; 2 The Instrumental Analysis Center of Shenzhen University, Shenzhen University, Shenzhen 518060, China; 3 Department of Molecular Medicine and USF Health Byrd Alzheimer’s Research Institute, Morsani College of Medicine, University of South Florida, Tampa, FL 33612, USA

**Keywords:** methyl-CpG-binding domain protein, intrinsically disordered protein, Glycine max, cryoprotective function, stress tolerance

## Abstract

Intrinsically disordered proteins (IDPs) are multifunctional due to their ability to adopt different structures depending on the local conditions. The intrinsically disordered regions of methyl-CpG-binding domain (MBD) proteins play important roles in regulating growth and development by interpreting DNA methylation patterns. However, whether MBDs have a stress-protective function is far from clear. In this paper, soybean GmMBD10c protein, which contains an MBD and is conserved in Leguminosae, was predicted to be located in the nucleus. It was found to be partially disordered by bioinformatic prediction, circular dichroism and a nuclear magnetic resonance spectral analysis. The enzyme activity assay and SDS-PAGE results showed that GmMBD10c can protect lactate dehydrogenase and a broad range of other proteins from misfolding and aggregation induced by the freeze–thaw process and heat stress, respectively. Furthermore, overexpression of GmMBD10c enhanced the salt tolerance of *Escherichia coli*. These data validate that GmMBD10c is a moonlighting protein with multiple functions.

## 1. Introduction

DNA methylation, a well-characterized epigenetic mark, can repress the transcription of genes and thereby affects many biological processes [1]. Most DNA methylation occurs at CpG dinucleotides. Methyl-CpG-binding domain (MBD) proteins primarily bind methylated CpG and interpret DNA methylation patterns by recruiting chromatin remodelers, transcription factors and histone deacetylases [2]. As a result of this function, MBD proteins are likely to regulate the growth and development of eukaryotes, including plants [3].

The first MBD protein was found in mice and was named MeCP2; it is a 53 kDa protein that contains a conserved 70 amino acid long MBD at its N-terminal end and a transcriptional repression domain (TRD) towards its C-terminus [4]. A structural analysis revealed that MeCP2 is a hybrid protein, which, in addition to ordered MBD, contains long intrinsically disordered regions, i.e., its polypeptide chain is partially unstructured [5]. Intrinsically disordered proteins (IDPs) are involved in many molecular processes because their flexible conformation allows them to bind multiple partners in different environments [6,7]. MeCP2 is a typical IDP in that it interacts with several partners, for example, transcriptional coactivator cAMP response element-binding protein, nuclear receptor corepressor 1 and DNA (cytosine-5)-methyltransferase 1 [8].

Plant MBD proteins are less well studied than those in animals [7], although genes encoding MBD proteins have been cloned from rice, Arabidopsis, wheat and maize [2,9]. MBD proteins can be divided into seven or eight classes based on their sequence similarity and phylogenetic relationships [10]. Although some AtMBDs (i.e., MBDs from Arabidopsis) are known to be involved in antisilencing, active demethylation and the regulation of flowering time, the various functions of most MBDs are far from clear [2].

IDPs have been found in the proteomes of a broad range of organisms [6,11]. They are characterized by structural instability and functional flexibility [12,13], serve as a foundation of the protein structure–function continuum concept [14,15,16,17] and can be involved in a number of critical processes in cells, such as control of the cell cycle, regulation of gene transcription, alternative splicing and signal transduction [11]. IDPs are also strongly associated with stress tolerance [13,18]; for example, a very large number of plant IDPs participate in defense against abiotic and biotic stresses by protecting client proteins, binding ions or promoting liquid–liquid phase separation [19,20,21]. Individual IDPs have also been shown to have more than one function, a property that has been termed “moonlighting” [22,23].

There are 21 MBD family proteins in *Glycine max* [9], most of which are predicted to be IDPs. In our previous study of the iTRAQ-labeled heat-stable proteome in soybean, the MBD protein GmMBD10c was found to be soluble after heating to 100 °C [19]. Curiously, high tolerance against various extreme conditions (such as high temperature and acidic or alkaline pH) is a characteristic feature of many IDPs [24,25,26,27,28]. Intriguingly, the expression level of GmMBD10c in stress-tolerant radicles was higher than that in stress-sensitive radicles [19]. However, whether GmMBD10c protein plays a protective role under stress conditions is still unclear. In this paper, the structural characteristics and protective function of GmMBD10c protein under stress conditions were analyzed.

## 2. Results

### 2.1. Bioinformatic Analysis of GmMBD10c Protein

**Figure 1:**
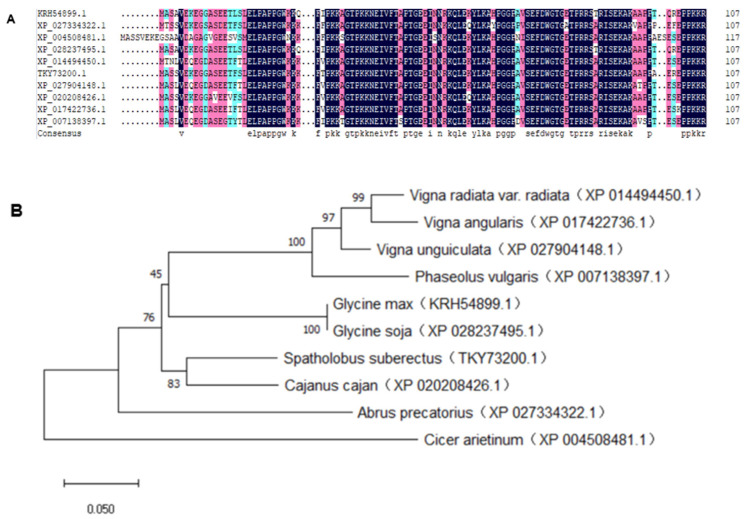
Sequence alignment and phylogenetic tree of the MBD domain of GmMBD10c and related proteins from various plant species. (**A**) The sequence alignment of GmMBD10c and related proteins using DNAMAN. Black color means that all amino acids are the same, and the color of red and green shows partial match amino acids. (**B**) Phylogenetic tree of GmMBD10c proteins constructed by MEGA.

DNAMAN version 6.0 (Lynnon Biosoft, USA) and Mega version X were used to conduct the sequence alignments and to generate a phylogenetic tree. GmMBD10c protein contains an MBD domain, which is located at residues 17–81 [9] and is similar to the MBDs of several MBD-containing proteins in other leguminous plants, such as *Glycine soja*, *Abrus precatorius* and *Phaseolus vulgaris* (Figure 1). This result implies that the MBD proteins are conserved in Leguminosae.

**Figure 2:**
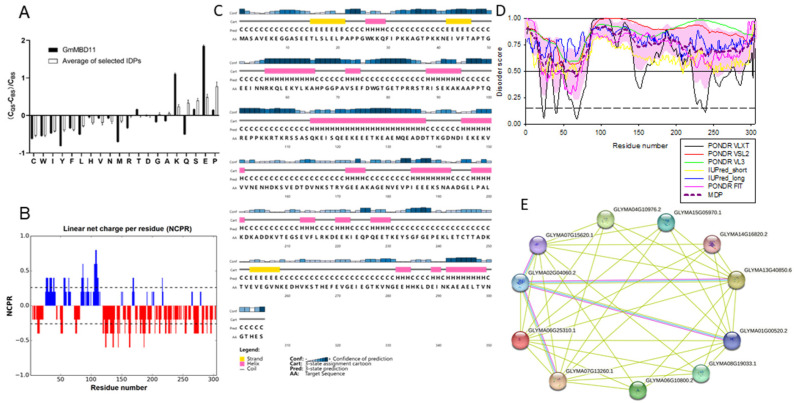
Bioinformatics analysis of the GmMBD10c protein. (**A**) The relative amino acid composition of GmMBD10c protein (black) and a set of disordered proteins selected from the DisProt database (white). (**B**) Charge distribution of GmMBD10c protein in CIDER. (**C**) Secondary structural prediction of GmMBD10c protein by PSIPRED. (**D**) Disorder prediction of GmMBD10c protein using RIDAO. (**E**) GmMBD10c-centered protein–protein interaction network generated by STRING.

The theoretical molecular weight of the GmMBD10c protein is 33.8 kDa. There are 74 negatively charged residues (Asp + Glu) and 47 positively charged residues (Arg + Lys) in the sequence (Figure 2A). The overall charge of GmMBD10c is -27 (Figure 2B). Overall, the amino acid sequence of the GmMBD10c protein is depleted in the major order-promoting residues (such as Cys, Trp, Ile, Tyr, Phe, Leu and Met) and is enriched in the major disorder-promoting residues (such as Lys, Ser, Glu and Pro). These peculiarities of the amino acid composition suggest that the GmMBD10c protein is likely to belong to the category of highly disordered proteins. The secondary structure of GmMBD10c, analyzed with PSIPRED software, was predicted to be mostly disordered, with some helices and turns (Figure 2C). Application of the RIDAO platform that integrates outputs of six commonly used disorder predictors (PONDR^®^ VLXT, PONDR^®^ VSL2, PONDR^®^ VL3, PONDR^®^ FIT, IUPred_short and IUPred_long) and generates the mean disorder profile (MDP) of the GmMBD10c sequence provided further support to the mostly disordered nature of this protein (Figure 2D). In fact, according to the MDP output, GmMBD10c is predicted to have a mean disorder score of 0.73 ± 0.11, which clearly classifies it as a highly disordered protein. Furthermore, IUPred2A indicated that GmMBD10c contains four long disorder-based protein interaction sites located at residues 1–99, 114–118, 131–221 and 232–305, indicating that almost 90% of the sequence of this protein can be engaged in interactions with protein partners. This observation is supported by the GmMBD10c-centered protein–protein interaction network generated by STRING (Figure 2E). This network includes 24 proteins connected by 105 interactions. The average node degree of this network is 8.75 and its clustering coefficient is 0.707, indicating that this network is tightly connected. Proteins from this network are involved in important biological processes, such as DNA demethylation (GO:0080111; *p* = 3 × 10^−11^), DNA methylation or demethylation (GO:0044728; *p* = 3 × 10^−11^), Histone H4 deacetylation (GO:0070933; *p* = 1.02 × 10^−10^), DNA metabolic processes (GO:0006259; *p* = 1.16 × 10^−10^) and seed maturation (GO:0010431; *p* = 4.52 × 10^−8^). Among the most significant biological functions of these proteins are methyl-CpG binding (GO:0008327; *p* = 1.56 × 10^−19^), NAD-dependent histone deacetylase activity (H3-K14 specific) (GO:0032041; *p* = 1.07 × 10^−9^), nucleic acid binding (GO:0003676; *p* = 2.39 × 10^−7^), poly(A)-specific ribonuclease activity (GO:0004535; *p* = 6.47 × 10^−7^) and ribonuclease activity (GO:0004540; *p* = 8.43 × 10^−6^). These proteins are cellular components of host cell nuclei (GO:0042025; *p* = 2.49 × 10^−24^), nuclei (GO:0005634; *p* = 2.80 × 10^−10^), histone deacetylase complexes (GO:0000118; *p* = 3.20 × 10^−8^), CCR4-NOT core complexes (GO:0030015; *p* = 9.13 × 10^−8^) and nucleoli (GO:0005730; *p* = 0.00027). In addition, the Plant-PLoc program predicted that GmMBD10c might be located in the nucleus, where it could regulate gene transcription.

### 2.2. Determination of Secondary Structure by Circular Dichroism

**Figure 3:**
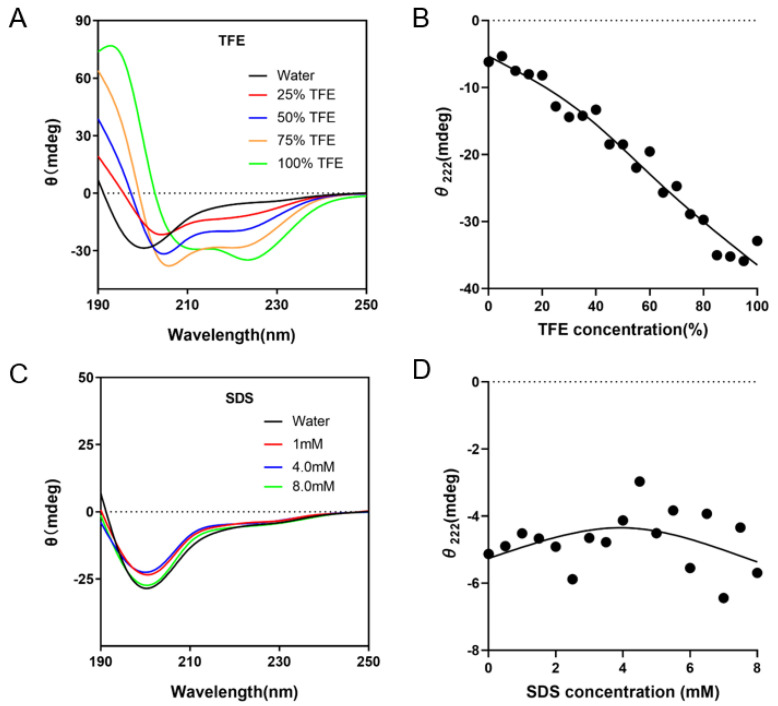
Circular dichroism analysis of the GmMBD10c protein in different solutions. The CD spectra of GmMBD10c protein were measured in aqueous solutions and in the presence of different concentrations of TFE (**A**) or SDS (**C**). The value of θ222 was plotted with the step-size of 5% of TFE (**B**) or 0.5 mM of SDS (**D**).

The far-UV CD spectra of GmMBD10c in water showed a deep minimum near 198 nm, which is indicative of a mostly disordered structure. When TFE was added to the solution, the far-UV CD spectra underwent dramatic changes, where one can see two minima at 208 nm and 222 nm and a peak at 192 nm, showing that TFE induces α-helical structure in GmMBD10c. In contrast, even at a concentration of 8 mM, SDS was unable to induce folding of GmMBD10c and this protein remained mostly unstructured (Figure 3). The proportion of helix, strand, turns and disorder in GmMBD10c was calculated using DICHROWEB. The results showed that GmMBD10c was 54.7% disordered in aqueous solutions, mainly consisting of random coils. In the presence of TFE, the helix content increased with increasing TFE concentration (Table 1). SDS is an anionic surfactant and therefore micelles formed by SDS are negatively charged. Since the overall charge of GmMBD10c is −27 (Figure 2B), it is unlikely that this protein would interact with negatively charged SDS micelles.

### 2.3. ^1^H-^1^D NMR Spectrum of the GmMBD10c Protein

**Figure 4:**
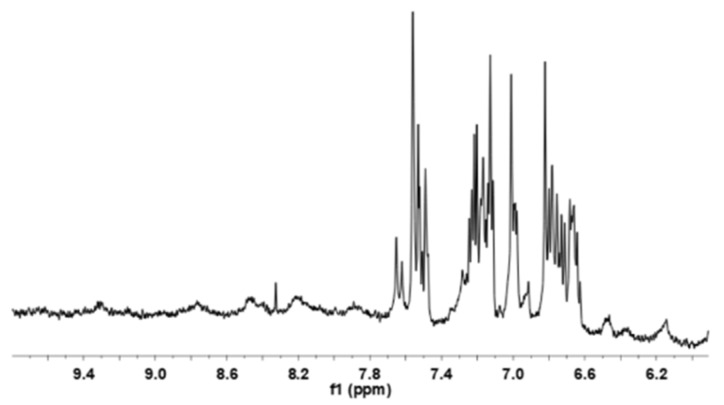
^1^D-^1^H NMR spectrum of GmMBD10c. The broad peaks in the amide region from 6.6 to 7.8 ppm show that GmMBD10c is a partially disordered protein.

To confirm the disordered structure of GmMBD10c, a ^1^H NMR spectrum was obtained and analyzed. The peaks in the amide region of the spectrum (from 6.6 to 7.8 ppm) are not well dispersed, similar to that of soybean LOC protein, another partially unstructured protein [29].Therefore, we conclude that GmMBD10c is partially disordered in aqueous medium (Figure 4).

### 2.4. GmMBD10c Preserves LDH Activity after Freeze–Thaw Treatment

**Figure 5:**
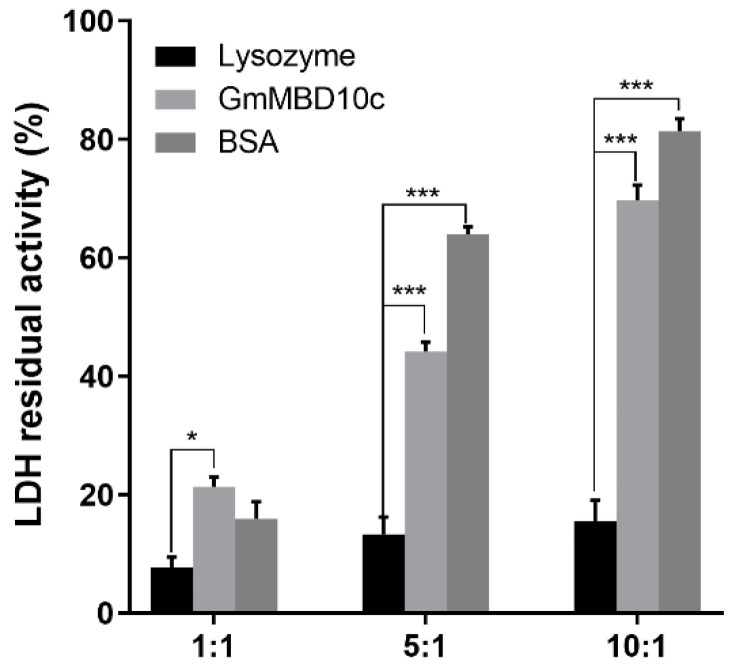
The protective effect of GmMBD10c protein on LDH under freeze–thaw conditions. The experiments were repeated three times. A non-parametric Kruskal–Wallis test was performed to evaluate whether there were significant differences among treatments. * *p* < 0.05 and *** *p* < 0.001.

We studied whether GmMBD10c could preserve the LDH activity after freeze–thaw treatment, which normally damages the enzyme. Our analysis revealed that the LDH activity dropped below 20% after freeze–thaw cycling in the absence of GmMBD10c. At a ratio of 10:1 (GmMBD10c/LDH), the LDH activity was maintained at approximately 70% of the non-stressed levels, which is much higher than the effects observed when the negative control, lysozyme, was used. The efficiency of GmMBD10c to maintain the LDH activity was comparable to that of BSA, which was used in these experiments as a positive control. This shows that GmMBD10c can efficiently protect LDH from freeze–thaw damage (Figure 5).

### 2.5. Thermoprotective Effect of GmMBD10c on the Proteome

**Figure 6:**
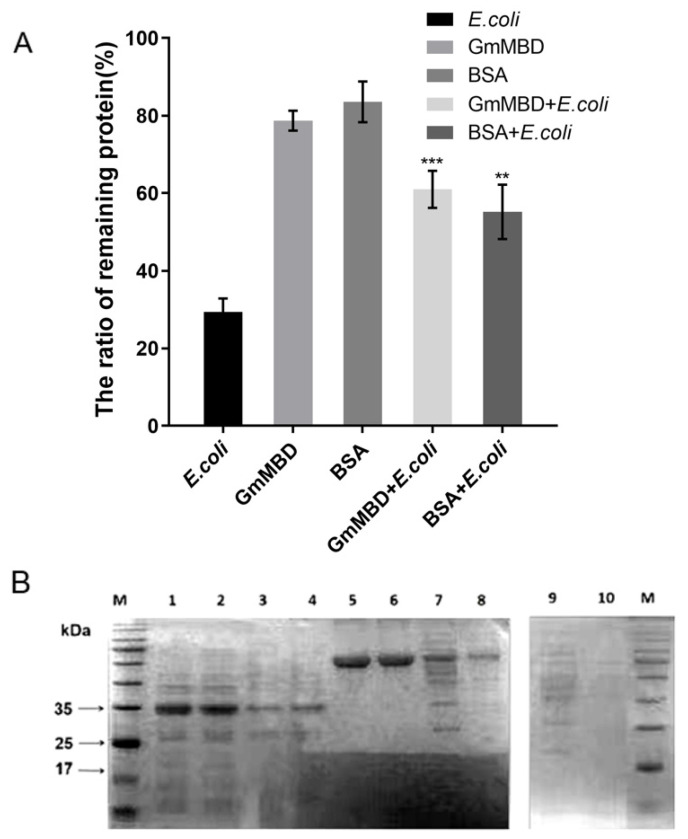
GmMBD10c protein protects *E. coli*’s proteome from heat denaturation. (**A**) The *E. coli* proteome was boiled at 100 °C for 2 min. The heat-stable proteome was quantity detected by a BCA assay, and the percentage of the remaining soluble protein was calculated. These experiments were repeated twice. A Shapiro–Wilk test showed that the data have a normal distribution. A student’s *t* test was performed between the *E.coli* proteome and GmMBD + *E.coli*, the results suggesting that two groups had statistically significant differences. ** *p* < 0.01 and *** *p* < 0.001. (**B**) The remaining soluble proteome in the supernatant was detected by SDS-PAGE. M, marker. Lanes 1, 3, 5, 7 and 9 are the purified GmMBD10c protein, GmMBD10c + proteome, BSA, BSA + proteome and proteome, respectively, and lanes 2, 4, 6, 8 and 10 are the boiled corresponding sample described above.

IDPs are thought to protect a broad range of proteins in the cell [30,31,32,33,34,35]. We tested this potential of GmMBD10c with the soluble proteome of *E. coli*, which was boiled in the presence of BSA or GmMBD10c. The results showed that nearly 40% of the proteome alone remained stable in the supernatant after boiling, whereas about 80% of the proteins remained soluble in the presence of GmMBD10c (Figure 6A). Furthermore, the SDS-PAGE pattern of the thermostable proteins was similar to that of the unheated proteome, indicating that the GmMBD10c protein is able to protect most of the proteins in *E. coli*’s proteome (Figure 6B).

### 2.6. GmMBD10c Increases Salt Tolerance in E. coli

**Figure 7:**
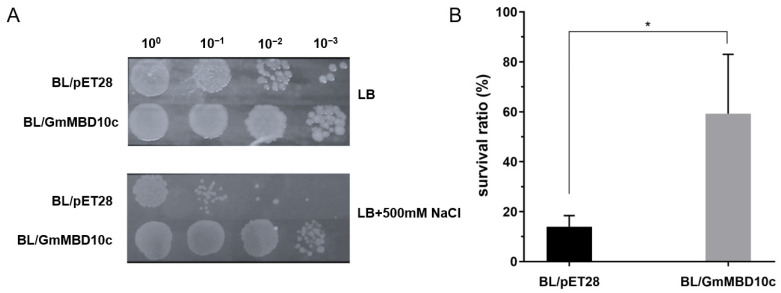
The spot assay and survival ratio of recombinants under 500 mM NaCl stress. (**A**) The growth performances of BL/GmMBD10c and BL/pET28 under normal or salt conditions were compared. Experiments were repeated three times and similar results were obtained. (**B**) The survival ratio assay was repeated three times and the significant differences were calculated by a student’s *t* test. * *p* < 0.05.

To determine the protective function of the GmMBD10c protein in cells under stress conditions, equal amounts of BL/pET and BL/GmMBD10c *E. coli* strains were serially diluted and spotted on LB or high NaCl medium. The spot assay showed that BL/pET and BL/GmMBD10c grew well in LB medium, suggesting that the expression of GmMBD10c does not adversely affect the growth of *E. coli* under favorable growth conditions. Under high salt conditions, however, the BL/GmMBD10c strain grew better than BL/pET (Figure 7A). In addition, quantitative tests showed that the survival ratios of BL/pET and BL/GmMBD10c were about 15% and 60% in the presence of 500 mM NaCl, respectively (Figure 7B). These results indicate that the overexpression of GmMBD10c can enhance the salt tolerance of *E. coli.*

## 3. Discussion

Proteins containing a disordered sequence of no less than 30 consecutive amino acids are defined as IDPs, and the unstructured sequence is called an intrinsically disordered region (IDR) [36]. Although lacking a well-defined conformation, IDPs are involved in many biological processes, such as post-translational modifications, conformational or allosteric control and extra/intracellular trafficking [37]. In an IDP, the IDRs are structurally plastic and may affect the stability and molecular function of the more ordered regions of the same protein [38]. The first identified MBD protein, MeCP2, is partially disordered, multifunctional and consists of six domains. The MBD-flanking domains (NTD and ID) are completely disordered and can alter the structure and function of the MBD domain [39]. Soybean GmMBD10c protein belongs to the class 1 MBD protein family and contains a high ratio of disorder-promoting amino acids. In water, GmMBD10c is largely disordered, with several disordered regions predicted in the region comprising aa 80–305. This high degree of disorder may provide the structural basis for the multifunctional nature of GmMBD10c.

A protein performing more than one function is called a moonlighting protein. Through evolution, proteins with a single function have acquired additional functions. Examples include some enzymes that can have additional non-enzymatic functions. ε-crystallin in duck eye lenses has almost as much enzymatic activity as LDH purified from duck hearts [22,40,41]. Protein moonlighting is beneficial to cells and organisms by coordinating multiple activities’ responses to changes in cellular conditions. The moonlighting protein performs different functions by binding to another protein or using different solvent-exposed surface areas [41]. The structural plasticity of IDPs allows them to play diverse roles in the growth and development of organisms [42]. They are also reported to be involved in stress responses by protecting client proteins or binding to membranes [43,44]. However, not all IDPs have this protective function [19,45]. Under stress conditions, most IDPs are stable in solution. Indeed, a key characteristic of IDPs is their ability to remain soluble after heating to 100 °C [19]. In this paper, the disordered GmMBD10c protein was shown to protect LDH and the *E. coli* proteome from freeze–thaw damage and heat stress, respectively. Overexpression of GmMBD10c was also able to enhance the salt tolerance of *E. coli*. The ability to protect proteins and enhance the salt tolerance in cells has not previously been reported for MBD proteins, but is likely IDR mediated. Thus, similar to other IDPs, GmMBD10c shows the characteristics of a moonlighting protein; it can protect sensitive proteins from stress damage as a result of its flexible structure as well as perform a methyl-CpG-binding role. This is another example of the diversity of functions exhibited by IDPs and adds to our understanding of the role of these fascinating proteins.

By the bioinformatical analysis, the GmMBD10c protein was predicted to be disordered and form a protein–protein interaction network with other proteins. Most IDPs can protect target proteins through the chaperone function, similar to BSA [46,47]. In addition, IDPs can play protective roles through a molecular shield, where they inhibit the aggregation of stress-sensitive proteins without binding to the target protein [48,49]. IDPs occupy the space around the client protein to prevent its misfolding by a degree of entropy transfer [48]. However, not all IDPs can protect client proteins, and the protective effect does not rely on the percentage of disordered regions [19]. Further research is needed to reveal the mechanisms of how the intrinsically disordered region plays a protective role in cells.

## 4. Materials and Methods

### 4.1. Strains

The soybean GmMBD10c gene (Glyma06g25310.1) was cloned and inserted into the pET28a vector. The resultant recombinant plasmid was transformed into *E. coli* BL21 to create the BL/GmMBD10c strain. There is a 6 × His-tag at the N-terminus of the fusion protein. The expression of fusion GmMBD10c protein was induced by 0.02 mM isopropyl-beta-D-thiogalactopyranoside (IPTG).

### 4.2. Bioinformatical Analysis

The primary structure of GmMBD10c was analyzed using CIDER at http://157.245.85.131:8000/CIDER/ (accessed on 10 November 2022) [50] and ProtParam at http://web.expasy.org/protparam/ (accessed on 10 November 2022) [51] to obtain basic information and the amino acid distribution of the protein. PSIPRED 4.0 [52] was used to analyze the protein’s secondary structure at http://bioinf.cs.ucl.ac.uk/psipred/ (accessed on 9 May 2023). The degree of protein disorder was analyzed using the web platform Rapid Intrinsic Disorder Analysis Online (RIDAO) [53], which integrates the outputs of six commonly used per-residue disorder predictors such as PONDR^®^ VLS2, PONDR^®^ VL3, PONDR^®^ VLXT, PONDR^®^ FIT, IUPred-Long and IUPred-Short [54,55,56,57,58,59]. Protein–protein interaction (PPI) networks were generated using the STRING (search tool for recurring instances of neighboring genes) platform at http://string-db.org/ (accessed on 11 May, 2023) [60]. The subcellular localization of the GmMBD10c protein was predicted at www.csbio.sjtu.edu.cn/bioinf/plant/ (accessed on 10 May 2022) [61]. Ten homologous MBD proteins were chosen by Blast and the phylogenetic tree was constructed by MEGA version X software from https://www.megasoftware.net/ (accessed on 19 January 2022) [62]. The sequence alignment was performed by DNAMAN 6.0 [63] from https://www.lynnon.com/downloads.html (accessed on 19 January 2022).

### 4.3. Circular Dichroism

The fusion GmMBD10c protein was purified with a 10 mL Ni-NTA column. An amount of 20 mM Tris-HCl containing a range of concentrations of imidazole from 40 to 200 mM was used to remove the impurity protein and obtain the GmMBD10c protein. The purified protein was desalted, freeze-dried and then dissolved in deionized water, TFE (with the step-size of 5%) or SDS (with the step-size of 0.5 mM) to a final concentration of 5 μM. The protein solution was subjected to circular dichroism spectroscopy(Jasco, Japan) in the ultraviolet range (250 nm to 190 nm) with readings taken at 1 nm intervals. The data were analyzed via Dichroweb to calculate the secondary structure content. Each experiment was repeated three times. The data of GmMBD10c in the presence of TFE (25%, 50% and 75%) or SDS (1 mM, 4 mM and 8 mM) were chosen to construct the spectrum. Additionally, the value of θ_222_ was plotted to show the structural changes of the GmMBD10c protein with an increasing concentration of TFE or SDS [29]. 

### 4.4. NMR Spectroscopy

The GmMBD10c protein was dissolved in 90% water and 10% deuterium oxide (D_2_O) to a final concentration of 100 μM. The experiment was performed with a cryogenic probe on a Bruker Avance III spectrometer at 500 MHz. The ^1^D^1^H spectrum was collected in the Zgesgp program. The data size was 32 K and the temperature was 23 °C and TopSpin 3.2 was used for analysis. The gradient excitation sculpting method was used for water suppression.

### 4.5. LDH Activity Measurement

Lactate dehydrogenase (LDH) is sensitive to freeze–thaw cycles and could be used to show the protection function of stabilizers [29]. The protection of the GmMBD10c protein in LDH in a freeze–thaw assay was performed according to reference [29]. In brief, the test protein was added to the LDH solution at a molar ratio of 1:1, 5:1 or 10:1 (test protein: LDH). The residual enzyme activity was calculated according to the formula:

Residual enzyme activity (%) = (A_340_ after freeze–thaw of the tested protein sample/ΔA_340_ before freeze–thaw) × 100%.

### 4.6. The Spot and Survival Ratio Assay

*E. coli* cultures were induced to express recombinant protein by treatment with 1 mM IPTG for 4 h. The concentration of these cultures was adjusted to OD_600_ = 0.8, then diluted serially (1:10, 1:100 and 1:1000). Ten microliters of each sample were dropped onto LB plates containing 0.02 mM IPTG and 500 mM NaCl. In addition, 100 μL samples were spread on the plates to count the colony number. After incubation at 37 °C for 48 h, photos of the spot assay plates were taken and the *E. coli* survival ratios were calculated as the number of plaques on the stress medium divided by the number of plaques on the LB medium.

### 4.7. ThermoStability of the E. coli Soluble Proteome

Soluble protein was extracted from *E. coli* cells, then quantified by a BCA assay. An amount of 1 g/L GmMBD10c or BSA was mixed with the proteome sample and incubated at 100 °C for 2 min. The soluble protein extract was used as a control. Samples were centrifuged at 10,000 rpm for 20 min at 4 °C. The protein in the supernatant was quantified again by a BCA assay.

### 4.8. Statistical Analysis

The LDH active assay was analyzed by a non-parametric Kruskal–Wallis test, and the other assay was analyzed by a student’s t test.

## Figures and Tables

**Table 1:** The calculated proportion of the secondary structure in GmMBD10c protein.

Solution	Helix (%)	Strand (%)	Turns (%)	Unordered (%)
Water	11.3	6.9	27.2	54.7
25% TFE	31.6	8.0	19.8	40.7
50% TFE	62.2	2.8	11.0	23.9
75% TFE	78.5	1.8	6.7	13.4
1 mM SDS	10.7	5.3	26.8	57.2
4 mM SDS	10.8	6.5	28.0	54.8
8 mM SDS	11.6	6.2	28.6	53.7

## Data Availability

Not applicable.

## Abbreviations

CD	Circular dichroism
IDP	Intrinsically disordered proteins
LDH	Lactate dehydrogenase
MDP	Mean disorder profile
MBD	Methyl-CpG-binding domain
NMR	Nuclear magnetic resonance
RIDAO	Rapid Intrinsic Disorder Analysis Online
SDS	Sodium dodecyl sulfate
TFE	Tetrafluoroethylene
TRD	Transcriptional repression domain
